# Accuracy analysis of dam deformation monitoring and correction of refraction with robotic total station

**DOI:** 10.1371/journal.pone.0251281

**Published:** 2021-05-06

**Authors:** Jianguo Zhou, Bo Shi, Guanlan Liu, Shujun Ju

**Affiliations:** 1 School of Civil Engineering, Architecture and Environment, Hubei University of Technology, Wuhan, Hubei, China; 2 Changjiang Spacial Information Technology Engineering Co., Ltd., Wuhan, Hubei, China; 3 School of Geodesy and Geomatics, Wuhan University, Wuhan, Hubei, China; 4 Guodian Dadu River Hydropower Development Co., Ltd., Chengdu, Sichuan, China; Al Mansour University College-Baghdad-Iraq, IRAQ

## Abstract

Robotic total stations have been widely used in continuous automatic monitoring of dam deformations. In this regard, monitoring accuracy is an important factor affecting deformation analysis. First the displacements calculation methods for dam deformation monitoring with total stations are presented, and the corresponding mean square error formulas are derived. Then for errors caused by atmospheric refraction, two correction methods are described. Simulations were conducted to compare the displacement accuracy calculated through different methods. It indicated that the difference between polar coordinate method and forward intersection is less than 0.5mm within around 400m’ monitoring range, and in such cases, the polar coordinate method is preferred, as only one total station is required. Refraction correction tests with observations from two dams demonstrated that both correction methods could effectively enhance the monitoring accuracy. For observation correction, correction through the closest reference point achieves better correction results.

## 1. Introduction

As one of the most important engineering infrastructures, reservoir dams play an essential role in flood control, power generation, and irrigation [1]. Safety cannot be ignored when operating dams, because if failures occur, they will have devastating consequence for people, property and the environment. We have witnessed several such cases in history [2]. To fully understand the health of a dam, dam safety monitoring is indispensable [3]. Dam safety monitoring runs through its whole lifecycle. During the planning phase, the overall plan of the safety monitoring system is designed with the list and layout of the monitoring equipment. And the instruments are installed or buried into the dam structure during the construction phase. In operation phase, periodic and special monitoring are carried out to assess the safety of the dam. Geodetic, geotechnical, and environmental sensors can be employed to monitor the deformation, internal stress, seepage, water level, etc [4]. Deformation monitoring is one of the most important aspects of dam safety monitoring [5]. With the development of geodetic surveying technology, geodetic sensors represented by level, total station, global navigation satellite system (GNSS) receiver, terrestrial laser scanner (TLS), and ground-based synthetic aperture radar (GB-SAR) have been applied to dam deformation monitoring [6].

Leveling is a reliable method for monitoring the vertical displacement and is currently extensively employed in dam deformation monitoring [7]. However, it is a time-consuming method. Hydrostatic leveling is an effective alternative for providing continuous automatic monitoring of the vertical displacement [8], but the deployment difficulties limit its monitoring range. GNSS technology does not require visibility among monitoring points. It can realize continuous three-dimensional displacement monitoring in all weather conditions, which has several advantages in dam deformation monitoring [9, 10]. However, the monitoring accuracy is highly affected by satellite visibility and the multipath effect. Terrestrial laser scanning technology has good application prospects in deformation monitoring since it can rapidly obtain a high-density point cloud of the dam and can achieve entire dam deformation analysis [11, 12]. Due to the slightly low accuracy of a single point acquired by TLS, for example about 10 mm when the TLS is located at a distance of about 100 m [13], highly accurate deformation analysis through modeling is still challenging. GB-SAR technology can provide long-distance, large-scale, and continuous spatial coverage of deformation results and its monitoring accuracy can reach 0.1mm [14]. Currently, it is used in dam monitoring [15, 16]. The main disadvantage of GB-SAR is that only deformation in the line of sight can be obtained. Furthermore, the equipment is expensive.

The total station is one of the most representative geodetic instruments, and it plays an important role in the field of deformation monitoring. It has been broadly employed in deformation monitoring and geological hazards monitoring of dams [17], bridges [18], tunnels [19], and other civil infrastructures. Especially with the improvement of accuracy and automation in the total station [20–22], the birth of the robotic total station (RTS) makes its advantages in continuous automatic monitoring of deformation more obvious. The monitoring accuracy of horizontal and vertical displacements is the key to the success of deformation analysis. Regarding deformation monitoring using total stations, the monitoring accuracy in tunnels has been analyzed and tested [23, 24]. However, dams are somewhat different from tunnels, and typically the monitoring range is broader. Therefore, it is necessary to analyze the monitoring accuracy in dams to guide the actual deployment of the dam deformation monitoring system based on robotic total stations. Moreover, dam deformation monitoring is more susceptible to atmospheric conditions, which may bring large errors to distance and angle observations collected by the total station due to refraction [25]. Atmospheric refraction affects all kinds of remote measurement systems [26, 27]. Some studies have employed meteorological sensors on-site to establish atmospheric models to rectify observations [28]. The main drawback of such methods is that additional meteorological sensors are needed. Following the introduction of the continuous automatic monitoring system of dam deformation with robotic total stations, this study presents mathematical models for obtaining the horizontal and vertical displacements and corresponding accuracy estimation formulas. As for the influence of atmospheric refraction, two approaches to correcting monitoring results without meteorological measurements are described, which are observation correction and coordinate correction. The monitoring accuracy of different monitoring models was analyzed and compared through simulation and relevant suggestions are given with a view to actual deployment. The performance of the two refraction correction methods was validated through observations from two dams.

## 2. Methodology

For a continuous remote unattended monitoring system of dam deformation based on robotic total stations, the main objective is to measure the displacements of the dam embankment and the slopes on both banks and to grasp the health status of the dam structure and its surroundings.

**Fig 1:**
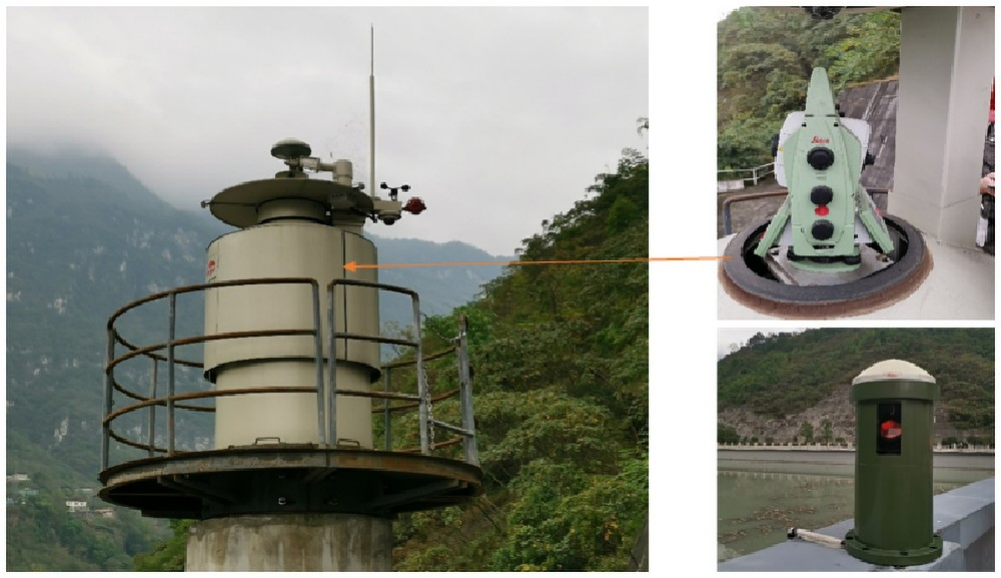
Dam deformation monitoring system based on robotic total stations.

Unlike indoor or underground monitoring tasks, weather is an important factor to consider in dam deformation monitoring. First, long-term sunlight, rain, and snow may corrode the total station if the setup is in the open air. Therefore, an observation room must be built and the total station has to be set up inside it. The observation room only opens the door for the total station to perform observation with specified monitoring frequency, which protects the instrument from harsh weather and wild animals. Then the station point should be equipped with meteorological sensors, which can be used to get the real-time meteorological data not only for correcting observations but also for determining whether the weather conditions are suitable for performing observing. In addition, as an unattended monitoring system, the site needs to be equipped with video surveillance and alarm systems for the sake of the safety consideration of the instruments. Besides, the prisms also should be shielded to ensure long-term usability as reflectors. Fig 1 shows a typical dam deformation monitoring system based on robotic total stations. For displacement calculations, plane displacement and vertical displacement are usually calculated separately. The plane displacement calculation adopts the polar coordinate method and forward intersection method. Furthermore, the vertical displacement is mostly calculated through trigonometric leveling.

### 2.1 Polar coordinate method

**Fig 2:**
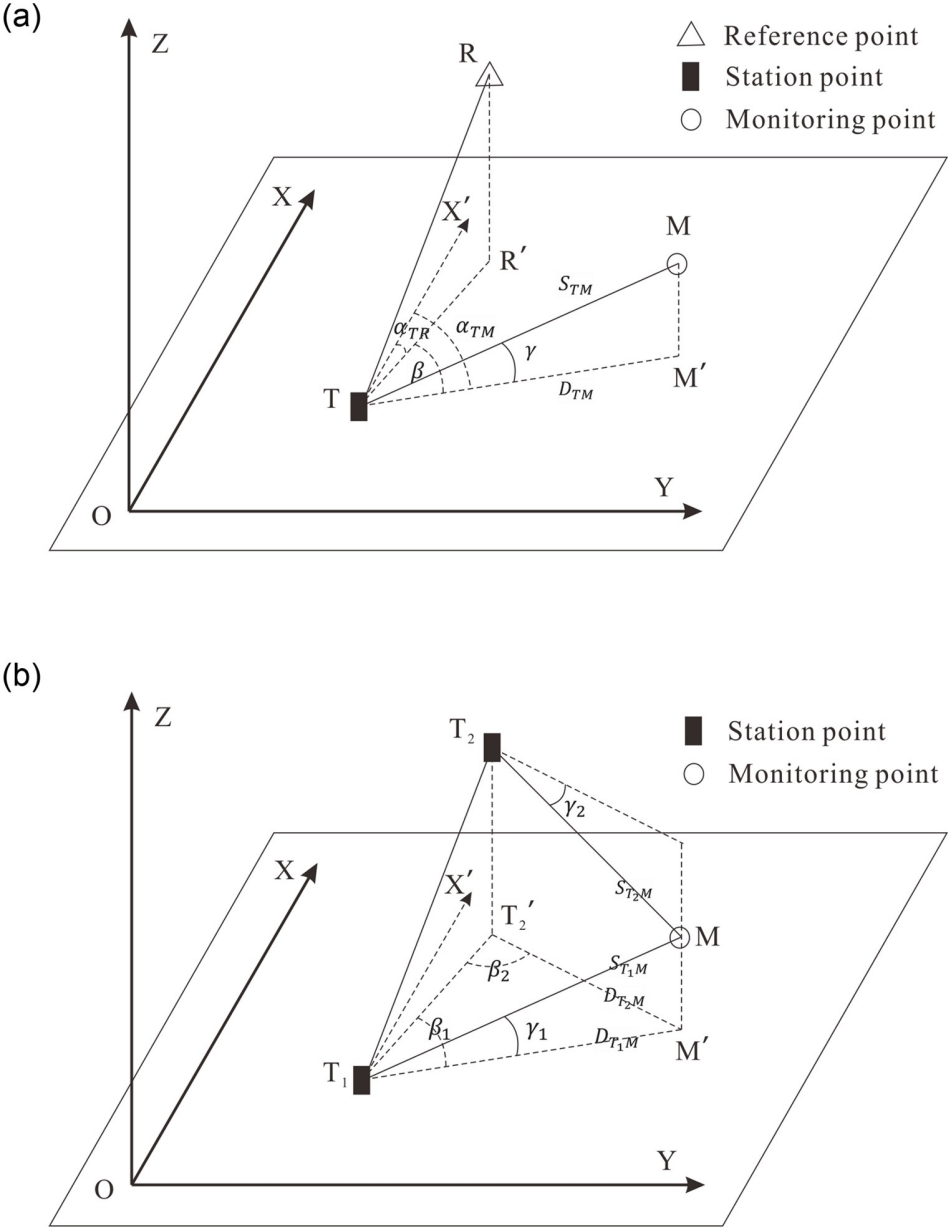
Dam deformation monitoring principle for plane coordinates with total station. (a) Polar coordinate method. (b) Forward intersection method.

Because the monitoring area of the total station is small, the effect of earth curvature is negligible when calculating the plane coordinates of monitoring points. For the polar coordinate method (Fig 2(a)), the total station is set up at station point *T* with known plane coordinates (*X*_*T*_, *Y*_*T*_). The station point should be stable during the monitoring period and should be convenient for the instrument observing monitoring points. Reference point *R* is usually located in a stable area, which is away from the deformation area, with known plane coordinates (*X*_*R*_, *Y*_*R*_). The function of the reference point is to help the orientation of the total station. For each monitoring epoch, the total station at station point *T* first sights the reference point *R* as backsight. Then it rotates to the monitoring point *M* to obtain the horizontal angle observation *β*, vertical angle observation *γ*, and slope distance observation *S*_*TM*_. With the known plane coordinates of point *T* and *R*, the azimuth of *TR*, denoted as *α*_*TR*_, can be calculated. Also, the plane coordinates of monitoring point *M*, denoted as (*S*_*M*_, *Y*_*M*_), can be computed with the following equations [29]:

$$\begin{array}{c} X_{M}=X_{T}+D_{TM}*\text{cos}\alpha_{TM}=X_{T}+S_{TM}*\text{cos}\gamma *\text{cos}\left(\alpha_{TR}+\beta \right)\\ Y_{M}=Y_{T}+D_{TM}*\text{sin}\alpha_{TM}=Y_{T}+S_{TM}*\text{cos}\gamma *\text{sin}\left(\alpha_{TR}+\beta \right) \end{array}$$ (1)

where *D*_*TM*_ stands for the horizontal distance from station point *T* to monitoring point *M*, and *α*_*TM*_ signifies the azimuth of *TM*.

Ignoring the coordinate errors of the reference point *R* and station point *T*, and without considering the impact of refraction and assuming that the accuracy of monitoring point *M* is only related to the observations, the mean square errors of monitoring point *M* in the X-axis and Y-axis are estimated using the rules of error propagation as follows:

$$\begin{array}{c} m_{X_{M}}=\sqrt{A^{2}{*\text{cos}}^{2}\left(\alpha_{TR}+\beta \right)+B^{2}{*\text{cos}}^{2}\left(\alpha_{TR}+\beta \right)+C^{2}{*\text{sin}}^{2}\left(\alpha_{TR}+\beta \right)}\\ m_{Y_{M}}=\sqrt{A^{2}{*\text{sin}}^{2}\left(\alpha_{TR}+\beta \right)+B^{2}{*\text{sin}}^{2}\left(\alpha_{TR}+\beta \right)+C^{2}{*\text{cos}}^{2}\left(\alpha_{TR}+\beta \right)} \end{array}$$ (2)

where $A=cos\gamma *m_{S_{TM}}$, $B=S_{TM}*\text{sin}\gamma *\frac{m_{\gamma }}{\rho }$, and ${C=S}_{TM}*cos\gamma *\frac{m_{\beta }}{\rho }.m_{S_{TM}}$, *m*_*β*_, and *m*_*γ*_ are the nominal precision of *S*_*TM*_, *β*, and *γ*, respectively, which are usually given by the manufacturer of total stations. *ρ* = 206265”, which is the constant used in the conversion of the radian into the degree.

The plane displacement $\Delta d_{M}^{i}$ of monitoring point *M* can be derived by comparing the *i*th monitoring epoch coordinates $\left(X_{M}^{i},Y_{M}^{i}\right)$ with the initial coordinates $\left(X_{M}^{0},Y_{M}^{0}\right)$:

$$\begin{array}{c} \Delta X_{M}^{i}=X_{M}^{i}-X_{M}^{0}\\ \Delta Y_{M}^{i}=Y_{M}^{i}-Y_{M}^{0}\\ \Delta d_{M}^{i}=\sqrt{{\left(\Delta X_{M}^{i}\right)}^{2}+{\left(\Delta Y_{M}^{i}\right)}^{2}} \end{array}$$ (3)

The mean square error of plane displacement $m_{{\Delta d}_{M}}$ can be calculated by combining Eqs (2) and (3) as follows:

$$m_{{\Delta d}_{M}}=\sqrt{2m_{X_{M}}^{2}+{2m}_{Y_{M}}^{2}}=\sqrt{2\left[{cos}^{2}\gamma *m_{S_{TM}}^{2}+\frac{S_{TM}^{2}*\left({sin}^{2}\gamma *m_{\gamma }^{2}+{cos}^{2}\gamma *m_{\beta }^{2}\right)}{\rho^{2}}\right]}$$ (4)

It can be observed from Eq (4) that, for the polar coordinate method, the accuracy of plane displacement of monitoring point *M* has nothing to do with the horizontal angle observation *β*. For a given instrument, the vertical angle and slope distance observations determine the monitoring accuracy.

### 2.2 Forward intersection method

For the forward intersection method, at least two total stations are needed to procure the plane displacement of the monitoring point [30]. Two total stations are set up at stable station points *T*_1_ and *T*_2_ with known plane coordinates $\left(X_{T_{1}},Y_{T_{1}}\right)$ and $\left(X_{T_{2}},Y_{T_{2}}\right)$, respectively (Fig 2(b)). In each monitoring epoch, the two total stations first sight each other as backsight, and then their telescopes rotate to monitoring point *M* to earn the corresponding horizontal angle *β*_1_, *β*_2_, vertical angle *γ*_1_, *γ*_2_, and slope distance $S_{T_{1}M},S_{T_{2}M}$ observations. As only the plane coordinates of monitoring point *M* are required here, the horizontal distance observation *D*_*TM*_ = *S*_*TM*_ * *cosγ* is applied for simplicity to construct the observation equation as follows:

$$\left\{\begin{array}{ll} D_{T_{1}M}+v_{D_{1}} & =\sqrt{{\left(X_{T_{1}}-{\hat{X}}_{M}\right)}^{2}+{\left(Y_{T_{1}}-{\hat{Y}}_{M}\right)}^{2}}\\ \beta_{1}+v_{\beta_{1}} & =\text{arctan}\frac{Y_{T_{1}}-{\hat{Y}}_{M}}{X_{T_{1}}-{\hat{X}}_{M}}-\alpha_{T_{1}T_{2}}\\ D_{T_{2}M}+v_{D_{2}} & =\sqrt{{\left(X_{T_{2}}-{\hat{X}}_{M}\right)}^{2}+{\left(Y_{T_{2}}-{\hat{Y}}_{M}\right)}^{2}}\\ \beta_{2}+v_{\beta_{2}} & =\alpha_{T_{2}T_{1}}-\text{arctan}\frac{Y_{T_{2}}-{\hat{Y}}_{M}}{X_{T_{2}}-{\hat{X}}_{M}} \end{array}\right.$$ (5)

where $v_{D_{1}},v_{\beta_{1}},v_{D_{2}},v_{\beta_{2}}$ represent observation residuals, ${\hat{X}}_{M},{\hat{Y}}_{M}$ indicate the unknown parameters (which should be determined) and coordinates of monitoring point *M*, respectively, and $\alpha_{T_{1}T_{2}},\alpha_{T_{2}T_{1}}$ stand for the azimuths of *T*_1_*T*_2_ and *T*_2_*T*_1_, which can be calculated based on the known coordinates of $\left(X_{T_{1}},Y_{T_{1}}\right)$ and $\left(X_{T_{2}},Y_{T_{2}}\right)$.

By writing the observation equations in matrix form, linearizing it using Taylor’s series, and truncating at the first order, we can reach the following equation:

$$L+v=f\left(\hat{X}\right)\approx f\left(X^{0}\right)+\frac{\partial f\left(X^{0}\right)}{\partial \hat{X}}\hat{x}$$ (6)

where **L** symbolizes the observations matrix;

**v** denotes the residuals matrix;

$\hat{X}$ signifies the unknown parameters matrix to be determined, which includes ${\hat{X}}_{M},{\hat{Y}}_{M}$;

**X**^**0**^ represents the approximate values of $\hat{X}$, which can be calculated according to the necessary observations; and $\hat{x}$ indicates the corrections for **X**^**0**^.

With the weight matrix **P** of observations, which can be obtained through the accuracy of the total station, the unknown parameters matrix $\hat{X}$, its cofactor matrix $Q_{\hat{X}}$, and the posteriori variance factor ${\hat{\sigma }}_{0}$ can be estimated according to the principle of least squares adjustment. Similar to the polar coordinate method, the plane displacement $\Delta d_{M}^{i}$ of monitoring point *M* can be derived by comparing with initial coordinates. The mean square error of plane displacement $m_{{\Delta d}_{M}}$ can be calculated as follows:

$$m_{{\Delta d}_{M}}={\hat{\sigma }}_{0}\sqrt{2\left(Q_{{\hat{X}}_{M}{\hat{X}}_{M}}+Q_{{\hat{Y}}_{M}{\hat{Y}}_{M}}\right)}$$ (7)

where $Q_{{\hat{X}}_{M}{\hat{X}}_{M}},Q_{{\hat{Y}}_{M}{\hat{Y}}_{M}}$ are the diagonal elements of $Q_{\hat{X}}$.

### 2.3 Vertical displacement calculation

**Fig 3:**
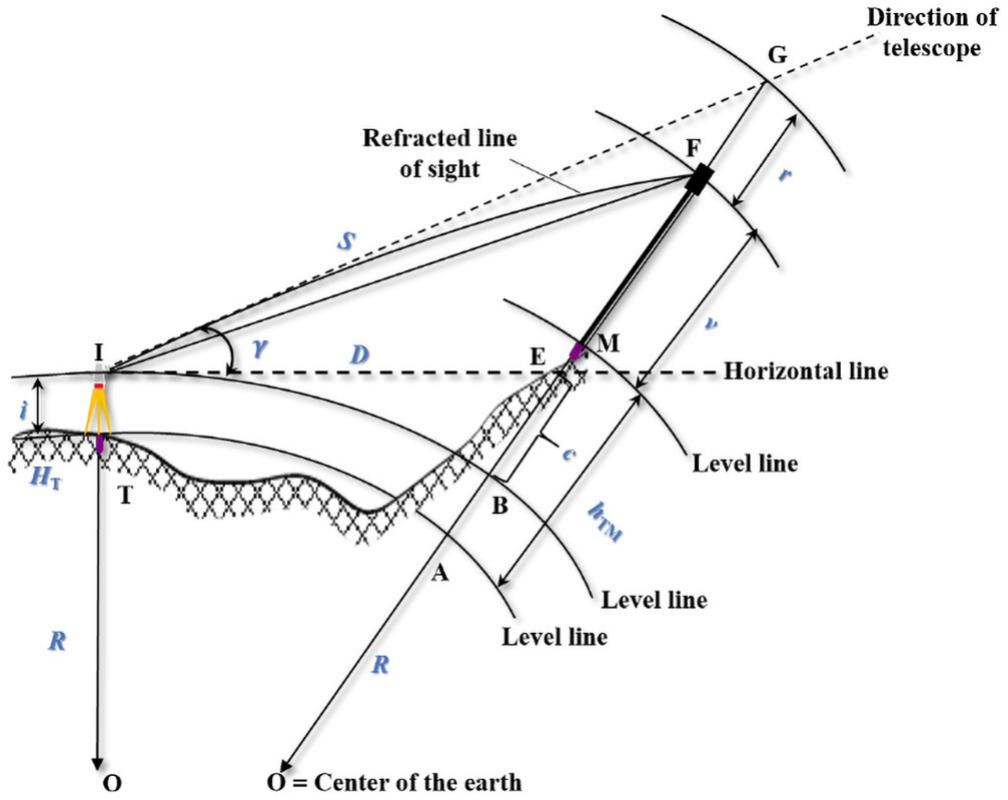
Dam deformation monitoring principle for height with total station.

When calculating the vertical displacement of the monitoring point with the total station, trigonometric leveling is utilized [31]. With no simultaneous reciprocal observations, the effect of curvature and refraction must be taken into account when calculating the vertical displacement of the monitoring point. As shown in Fig 3, the height of stable station point *T* is known as *H*_*T*_, and the total station at station point *T* sights the target *F* at monitoring point *M* to get the slope distance *S* and vertical angle *γ* observations in each monitoring epoch. Although the line of sight is refracted to target *F*, the telescope points to *G*, thus, *S* * *sinγ* = *EG* requires a correction for refraction *r* = *FG*. The effect of curvature *c* = *BE* is the difference between the horizontal line and level line crossing the instrument center *I* at the monitoring point *M*. The height of the instrument and target are expressed as *h*_*i*_ and *h*_*v*_, respectively. Then the height *H*_*M*_ of the monitoring point *M* can be computed as follows:

$$\begin{array}{ll} H_{M} & =H_{T}+h_{TM}\\ & =H_{T}+S*sin\gamma +c+h_{i}-r-h_{v} \end{array}$$ (8)

Usually, the effect of curvature and refraction can be approximately computed as $c=\frac{D^{2}}{2R}$ and $r=\frac{KD^{2}}{2R}$. Here, *D* symbolizes the horizontal distance, *R* represents the radius of the Earth, and *K* stands for the coefficient of refraction. By comparing the heights of *M* with the initial value, the vertical displacement $\Delta h_{M}^{i}$ can be calculated. For a continuous automatic monitoring system, the instrument and monitoring targets are fixed. The changes of horizontal distance *D* are very small relative to the radius of the Earth *R*. Then the height of the instrument *h*_*i*_ and target *h*_*v*_ and the effect of curvature *c* can be neutralized when calculating the vertical displacement $\Delta h_{M}^{i}$. That is, they do not constitute factors that affect the accuracy of $\Delta h_{M}^{i}$. However, the coefficient of refraction *K* is affected by the meteorological conditions, by the morphology of the ground, and by other complex factors. It can change substantially during different monitoring epochs. Therefore, the effect of refraction needs to be corrected when calculating the vertical displacement to keep monitoring accuracy. Assuming the effect of refraction has been corrected, the mean square error of vertical displacement $m_{{\Delta h}_{M}}$ can be calculated based on Eq (9):

$$m_{{\Delta h}_{M}}=\sqrt{2\left({sin}^{2}\gamma *m_{S}^{2}+S^{2}*{cos}^{2}\gamma *\frac{m_{\gamma }^{2}}{\rho^{2}}\right)}$$ (9)

## 3. Refraction correction

Affected by changes in temperature, humidity, and air pressure, the distribution of atmospheric density in the monitoring area is usually inhomogeneous in both vertical and horizontal planes. Especially for large hydropower dams built in the alpine-gorge area, topographic factor makes the meteorological conditions very complex, resulting in an extremely inhomogeneous distribution of atmospheric density. Then for long-term continuous automatic monitoring of dam deformation with robotic total stations, the refraction caused by uneven atmospheric density has a significant effect on the angle and distance measurements. Although reciprocal observing can eliminate the refractive error, it is not practical for automatic monitoring. It is essential to find a way to rectify the effect of refraction to retain the monitoring accuracy. The conventional method is to establish a model of the atmosphere to describe the change of the refraction index in space. And together with angle and distance measurements, the temperature, humidity, and air pressure are measured simultaneously and used to carry out corrections according to the certain atmosphere model. The deficiency of the atmosphere model based correction method is that high accuracy meteorological sensors are required. Moreover, meteorological measurements at the station point do not reflect the atmospheric conditions along the whole line. Instead, by using the known stable points as a reference, the measurements towards monitoring points can be corrected without meteorological data. The principle of which is similar to that of GNSS differential positioning.

When deploying the deformation monitoring reference net, some stable points are chosen and constructed to cover the monitoring area, which can be used for correction of observations. The fixed distances and angles between the station point and these points are treated as references. In each monitoring epoch, the observations from station point to monitoring points and those reference points are obtained in sequence. The differences between the fixed values and observations towards reference points are considered to be predominantly caused by changes in meteorological conditions. As one monitoring epoch conducted with a robotic total station takes a short time, it is assumed that the influence of meteorological conditions on observations towards reference points is similar to that of monitoring points. And those differences for reference points can be employed to rectify observations towards monitoring points with proper models to eliminate the influence of refraction. Depending on the mode of correction, the correction can be conducted on distance and angle observations, or the coordinates are directly rectified.

### 3.1 Distance correction

In monitoring epoch *i*, the total station at station point *T* obtains the distance observation *d*(*r*, *i*) towards reference point *R*. As the station point and reference point are considered as stable with known coordinates, the distance between them is fixed as *d*(*r*). Moreover, the difference between *d*(*r*) and *d*(*r*, *i*) can be thought to be caused by changes in meteorological conditions. The distance correction coefficient Δ*d*(*r*, *i*) can be calculated as follows:

$$\Delta d\left(r,i\right)=\frac{d\left(r,i\right)-d\left(r\right)}{d\left(r,i\right)}$$ (10)

If the raw distance observation *d*(*m*, *i*) from station point *T* to monitoring point *M* in the same monitoring epoch is measured, the corrected distance $\hat{d}\left(m,i\right)$ between them is:

$$\hat{d}\left(m,i\right)=d\left(m,i\right)-\Delta d\left(r,i\right)*d\left(m,i\right)$$ (11)

### 3.2 Horizontal angle correction

In deformation monitoring, the deformation value is generally determined relative to the first monitoring epoch. Then the horizontal direction measurement *β*(*r*) towards the reference point *R* in the first monitoring epoch can be taken as reference. The difference Δ*β*(*r*, *i*) between the measurement *β*(*r*, *i*) in monitoring epoch *i* and *β*(*r*) can be derived as:

$$\Delta \beta \left(r,i\right)=\beta \left(r,i\right)-\beta \left(r\right)$$ (12)

The difference Δ*β*(*r*, *i*) is chiefly caused by the change in zero direction of the horizontal circle and the atmospheric refraction in the horizontal plane, which has the same impact on horizontal angle observation *β*(*m*, *i*) towards monitoring point *M* in the same monitoring epoch. Δ*β*(*r*, *i*) needs to be subtracted from *β*(*m*, *i*) for corrected horizontal angle measurement $\hat{\beta }\left(m,i\right)$:

$$\hat{\beta }\left(m,i\right)=\beta \left(m,i\right)-\Delta \beta \left(r,i\right)$$ (13)

### 3.3 Height difference correction

The main function of the vertical angle is to calculate the height difference between points. Hence, we consider the correction for height difference instead of the vertical angle. The height difference between stable station point *T* and reference point *R* is fixed as *h*(*r*), and the height difference *h*(*r*, *i*) can be calculated based on the distance observation *d*(*r*, *i*) and vertical angle observation *γ*(*r*, *i*) in monitoring epoch *i*. The difference between *h*(*r*, *i*) and *h*(*r*) is largely caused by curvature and refraction, and the correction coefficient *C* is usually derived as follows:

$$C=\frac{h\left(r\right)-h\left(r,i\right)}{d^{2}\left(r,i\right){cos}^{2}\gamma \left(r,i\right)}$$ (14)

As each monitoring epoch takes a short time, it is believed that the curvature and refraction have the same influence on both reference point *R* and monitoring point *M*. Then based on the distance observation *d*(*m*, *i*) and vertical angle observation *γ*(*m*, *i*) towards monitoring point *M* in monitoring epoch *i*, the corrected height difference $\hat{h}\left(m,i\right)$ can be calculated as follows:

$$\hat{h}\left(m,i\right)=h\left(m,i\right)+Cd^{2}\left(m,i\right){cos}^{2}\gamma \left(m,i\right)$$ (15)

After rectifying the observations towards monitoring points, the coordinates of monitoring points can be calculated, and corresponding displacements can be derived.

### 3.4 Coordinate correction

Instead of correcting observations, coordinate correction utilizes uncorrected observations to calculate the coordinates of reference points first. The differences between calculated coordinates and known coordinates are considered as a result of refraction. Then the differences can be used to rectify the coordinates of monitoring points calculated with raw observations under a proper assumption.

For reference point *R* with known coordinates (*X*_*R*_, *Y*_*R*_, *H*_*R*_), the approximate coordinates (*X*_*R*_(*i*), *Y*_*R*_(*i*), *H*_*R*_(*i*)) in monitoring epoch *i* can be calculated with raw observations from station point *T* and principles mentioned in section two. The differences between them, which are thought to be principally caused by refraction, can be calculated as:

$$\begin{array}{ll} \Delta X_{R}\left(i\right) & =X_{R}\left(i\right)-X_{R}\\ \Delta Y_{R}\left(i\right) & =Y_{R}\left(i\right)-Y_{R}\\ \Delta H_{R}\left(i\right) & =H_{R}\left(i\right)-H_{R} \end{array}$$ (16)

In the same way, the approximate coordinates (*X*_*M*_(*i*), *Y*_*M*_(*i*), *H*_*M*_(*i*)) of monitoring point *M* in the same monitoring epoch can be calculated with raw observations towards it. And the differences calculated in Eq (16) are employed to rectify the approximate coordinates of monitoring point *M* and hence to get the corrected coordinates $\left({\hat{X}}_{M}\left(i\right),{\hat{Y}}_{M}\left(i\right),{\hat{H}}_{M}(i)\right)$.

## 4. Data analysis

### 4.1 Accuracy analysis

The accuracy of displacement monitoring for dams using total station was analyzed through simulations. First, the monitoring accuracy of horizontal displacement using polar coordinate and forward intersection methods was analyzed and compared. Then the monitoring accuracy of vertical displacement with trigonometric leveling was analyzed. In the simulation, the ranging accuracy was set as *m*_*S*_ = 0.6mm + 1 × 10^−6^S and horizontal and vertical direction measurements accuracy were set as *m*_*β*_ = *m*_*γ*_ = 0.5”, which was consistent with the commonly used Leica Nova TM50 robotic total station in dam deformation monitoring. Note that the effect of refraction was not taken into consideration here.

**Fig 4:**
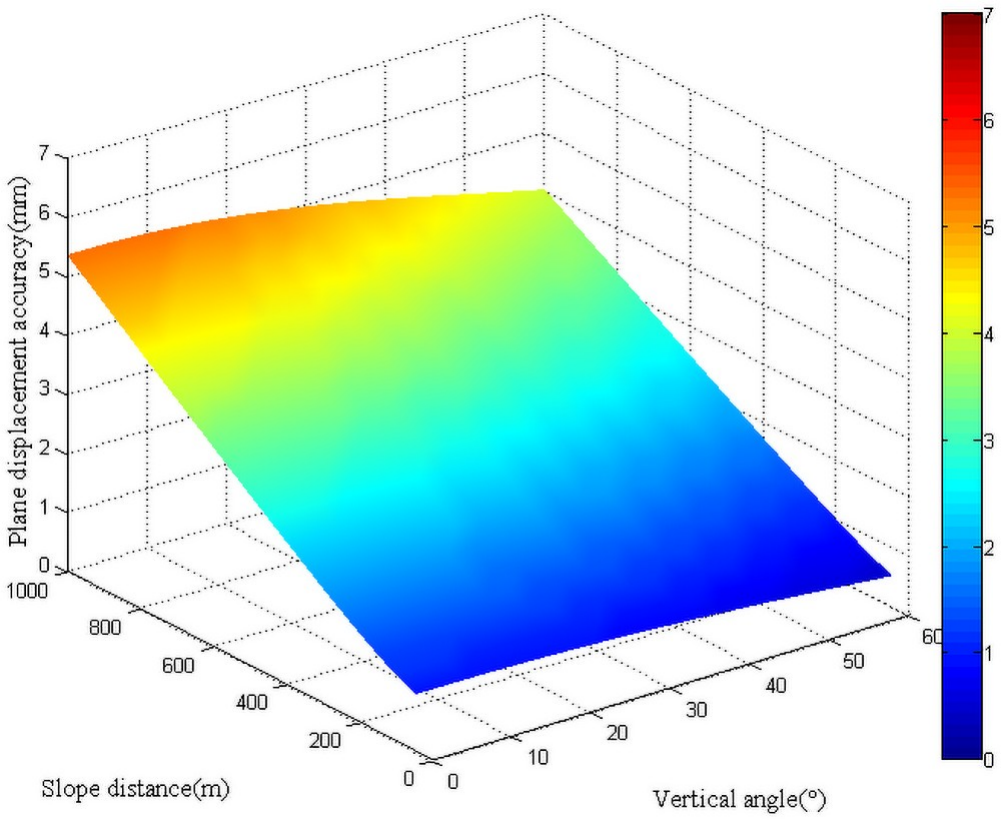
Plane displacement accuracy with the polar coordinate method.

For the polar coordinate method, the accuracy of plane displacement of the monitoring point was mainly affected by vertical angle and slope distance observations when the instrument was given according to previous theoretical derivation. Then the mean square errors of plane displacements under different vertical angles and slope distances were analyzed. The vertical angle changed from 0° to 60°, and the slope distance varied between 0 and 1000 m (Fig 4). It can be observed from the figure that when the vertical angle was fixed, the mean square error of plane displacement increased with the slope distance. To ensure the accuracy of plane displacement of the monitoring point, it was necessary to limit the slope distance from the station point to the monitoring point. When the slope distance remained fixed, the mean square error of plane displacement showed a decreasing trend with the increase of the vertical angle. This was because the horizontal distance diminished with the increase of the vertical angle. However, for actual monitoring, it is not recommended to increase the vertical angle to enhance the accuracy of plane displacement since a larger vertical angle will bring about greater atmospheric refraction for observations. Because of the symmetry, the accuracy of plane displacement when the vertical angle changed between 0° and -60° was consistent with that when the vertical angle varied from 0° to 60°.

**Fig 5:**
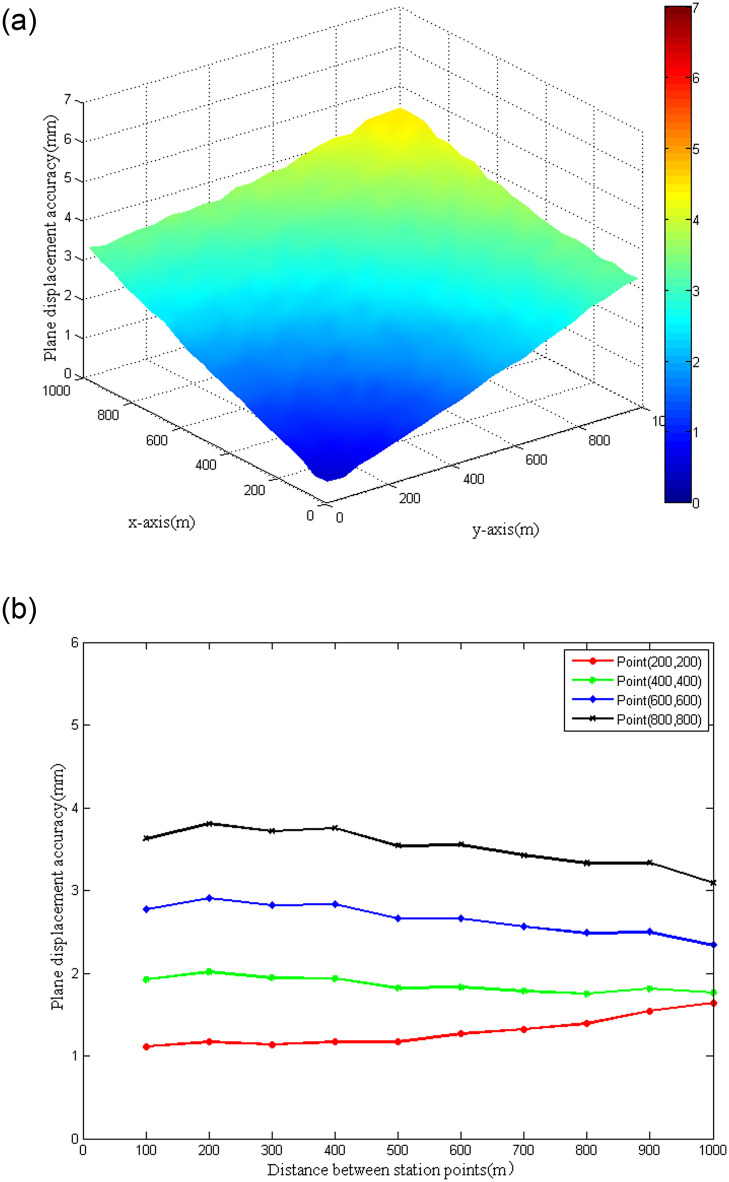
Plane displacement accuracy with the forward intersection method. (a) 100 m between station points. (b) Different distances between station points.

For the forward intersection method, when analyzing the accuracy of plane displacement of the monitoring point, the simulation scenario was set as follows: the distance between the two station points *T*_1_ and *T*_2_ was 100 m, and the corresponding coordinates were (0, −50) and (0, 50). According to the nominal accuracy of the Leica Nova TM50 robotic total station, the horizontal angle and distance observations between station points and the monitoring point were simulated. Then the plane displacement of the monitoring point was estimated and the accuracy was evaluated by the principle of least squares adjustment. Taking into account the symmetry of the observations, only the mean square errors of plane displacements for monitoring points located in the first quadrant were evaluated. Fig 5(a) indicates that if the midpoint of the two station points is considered as a reference, the mean square errors of plane displacements will almost be consistent when the distances from monitoring points to the reference point are equal. The mean square error of plane displacement for the monitoring point increased with the increase of the distance to the reference point. To further understand the influence of the distance between station points on the accuracy of plane displacements of monitoring points, four representative monitoring points ((200, 200), (400,400), (600, 600) and (800, 800)) were chosen for analysis. Taking the origin as the midpoint, the distance between the two station points varied from 100 m to 1000 m along the y-axis with an interval of 100 m. Fig 5(b) reveals that the accuracy differences in the plane displacement of the four representative monitoring points at different station point intervals did not exceed 0.5 mm. It could be concluded that the distance between station points had little effect on the accuracy of plane displacement. The above analysis results provided references for point deployment in actual dam monitoring.

**Fig 6:**
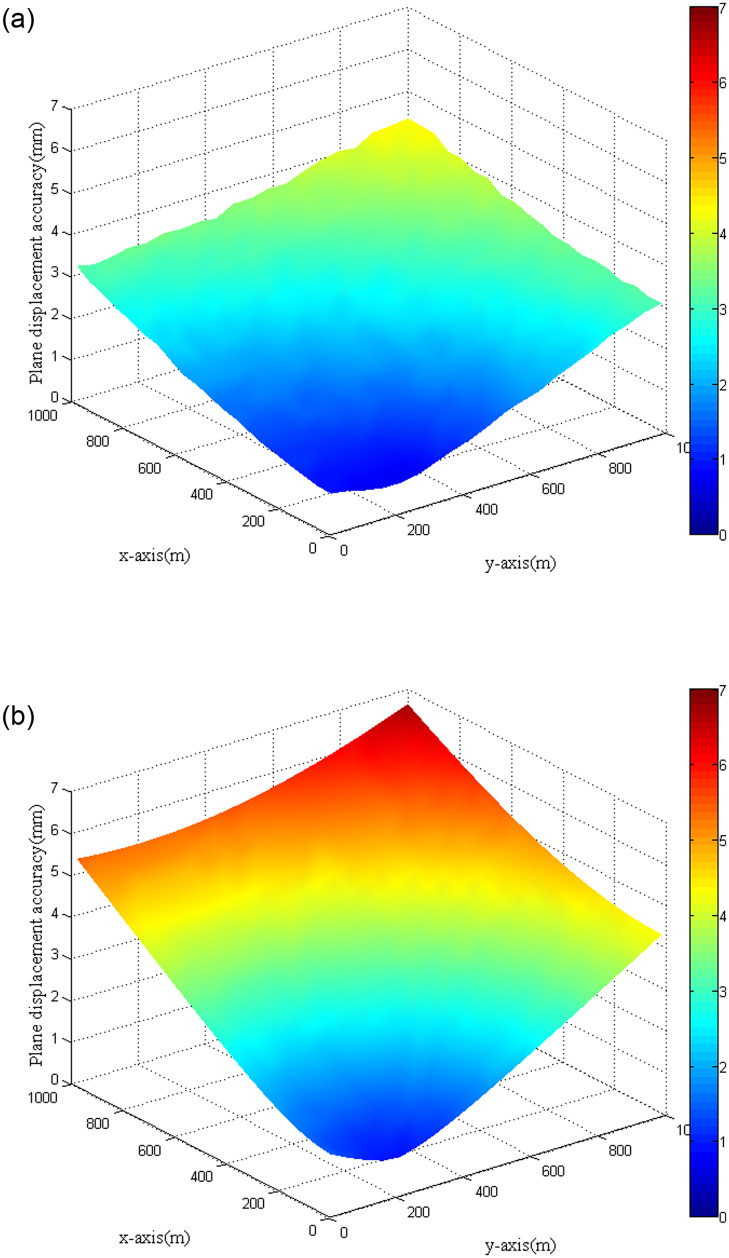
Plane displacement accuracy comparison. (a) Forward intersection method. (b) Polar coordinate method.

To compare the accuracy of the polar coordinate method and forward intersection method in calculating the plane displacements of monitoring points, the simulation was carried out. The setting of the simulation scenario was similar to the previous simulation. Here the distance between the two station points *T*_1_ and *T*_2_ was set to be 400 m, and the corresponding coordinates were (0, −200) and (0, 200). Then the mean square errors of plane displacements for monitoring points located in the first quadrant were calculated. To keep the consistency of the comparison, the vertical angle was set as *γ* = 0 for the polar coordinate method, which meant that the slope distance was equal to the horizontal distance. From the previous analysis, it could be known that the mean square error of plane displacement calculated with the polar coordinate method increased with the increase of the distance from the monitoring point to the station point. Then the station point *T*_2_ was selected for the polar coordinate method as it was closer to monitoring points located in the first quadrant. Fig 6(a) shows the mean square errors of plane displacements calculated with the forward intersection method, which further proved that the distance between station points had little impact on the accuracy of plane displacement because the result was similar to Fig 5(a). Fig 6(b) displays the mean square errors of plane displacements calculated with the polar coordinate method. It can be found from the figures that the accuracy of plane displacement obtained by the forward intersection method was better than that of the polar coordinate method. Moreover, it is seen that the gap increased as the distance from the station point *T*_2_ to the monitoring point increased, with a maximum of more than 2 mm. On the whole, the advantage of the polar coordinate method was that only one total station was needed to conduct the monitoring work. Hence, it was better to be applied to situations where the distances between the station point and monitoring points were short. The forward intersection method required the participation of multiple total stations. In this method, the mean square error was positively correlated with the distance from the monitoring point to the reference point. It mostly suited situations that monitoring points were far from station points and high monitoring accuracy was required.

**Fig 7:**
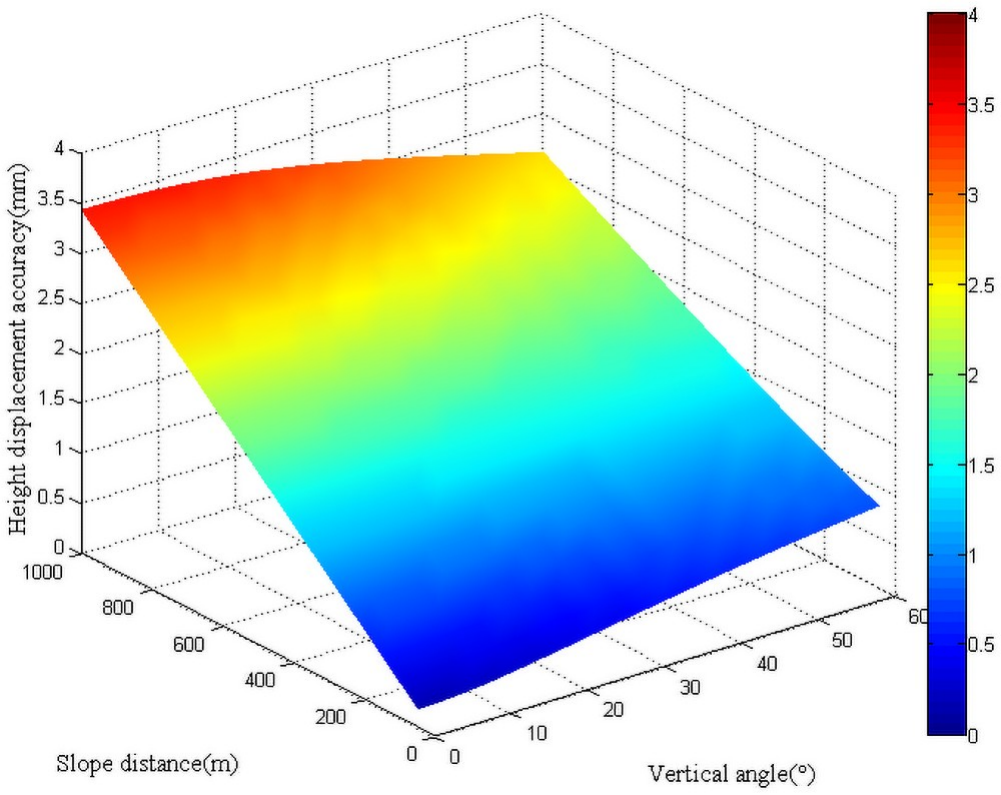
Vertical displacement accuracy with trigonometric leveling.

When trigonometric leveling was applied to calculate the vertical displacement of the monitoring point, the accuracy of vertical displacement was predominantly affected by vertical angle and slope distance observations when the effect of refraction was not taken into consideration. Then the mean square errors of vertical displacements under different vertical angles and slope distances were analyzed. The vertical angle changed from 0° to 60°, and the slope distance varied between 0 and 1000 m (Fig 7). It can be observed from the figure that when the vertical angle was fixed, the mean square error of vertical displacement increased with the slope distance. When the slope distance was short (less than 400 m), the mean square error of vertical displacement was positively correlated with the vertical angle. However, when the slope distance was large, the mean square error of vertical displacement exhibited a decreasing trend with the increase of the vertical angle. The same result could be derived when the vertical angle varied from 0° to -60°. For actual monitoring, the vertical angle needs to be limited to reduce the effect of refraction.

### 4.2 Correction tests

To verify the performance of refraction correction, two hydroelectric dams called Zhentouba I and Dagangshan in the Dadu River basin located in Sichuan province, China, were chosen for tests and analysis. According to the deployment of the dam deformation monitoring system, the observation correction test was conducted in Zhentouba I hydropower station, and the coordinates correction test was carried out in the Dagangshan hydropower station.

**Fig 8:**
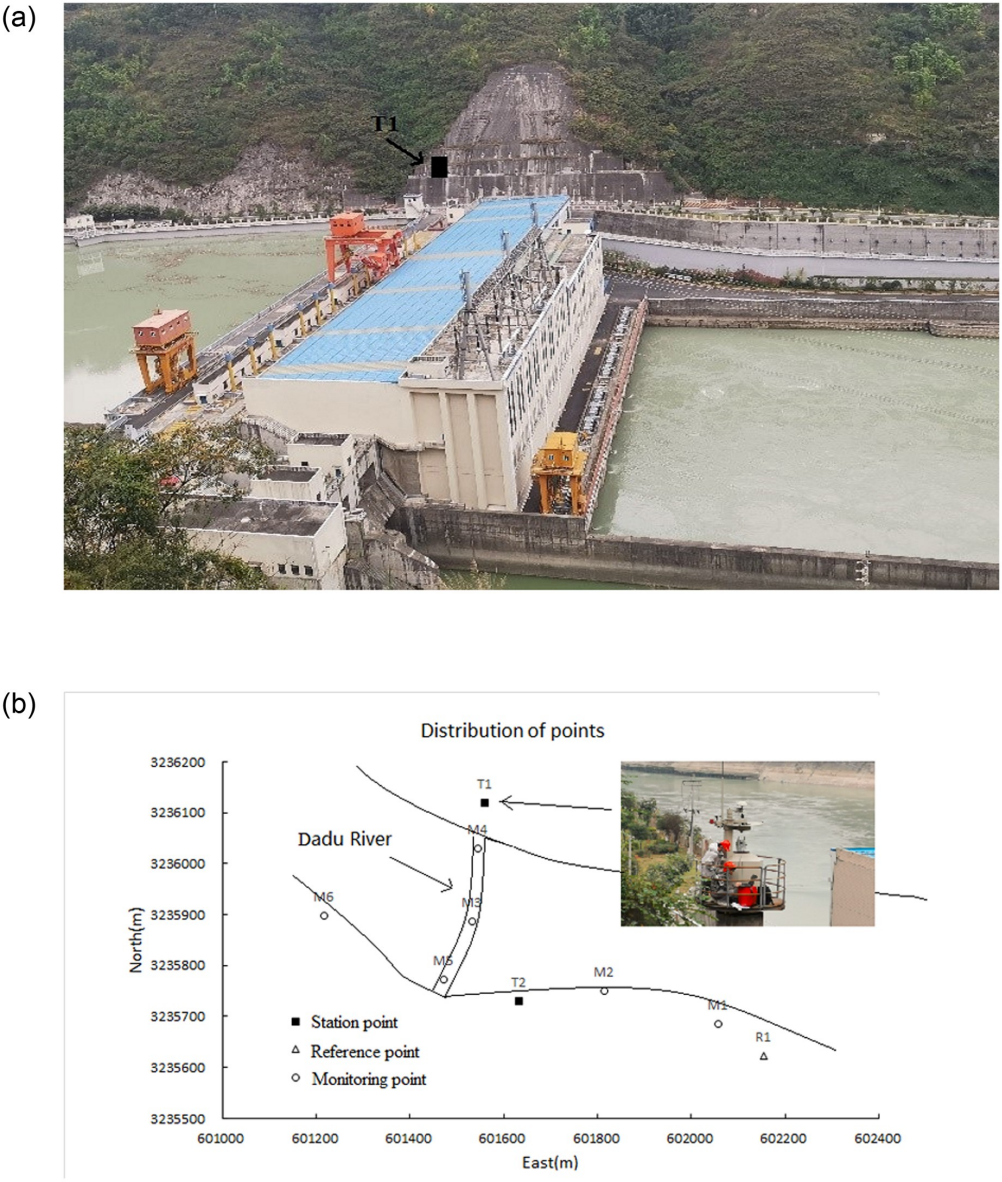
Refraction correction test in Zhentouba I hydropower station. (a) Zhentouba I hydroelectric dam. (b) Distribution of points in test.

The Zhentouba I hydroelectric dam is a concrete gravity dam, located in the middle and lower reaches of the Dadu River. As shown in Fig 8(a), the maximum dam height is 86.5 m, the elevation of the dam crest is 626.5m, and the total length of the dam crest is 317.55 m. To ensure the safety operation of the dam, an automated monitoring system of deformation was established using robotic total stations to monitor the displacements of the dam body and the slopes on both banks. When conducting the observation correction test affected by refraction, the established station point, reference point, and monitoring points were unitized. The Leica Nova TM50 robotic total station (*m*_*s*_ = 0.6 mm + 1 × 10^−6^S, *m*_*β*_ = *m*_*γ*_ = 1”) was set up at known station point *T*_1_ on the left bank (Fig 8(b)). The known reference point *R*_1_ far from the monitoring area on the right bank was selected as backsight, and raw distance and angle observations towards monitoring points *M*_1_ ~ *M*_6_ were obtained in sequence. Then the polar coordinate method was used to calculate the coordinates of monitoring points. At the same time, the observations towards the reference point *R*_1_ were utilized to correct the observations towards monitoring points in the same monitoring epoch. The corresponding coordinates were calculated with corrected observations. The whole test lasted about 17 hours from morning to evening. A total of 17 monitoring epochs were conducted with a one-hour interval. During the test, it was presumed that the monitoring points remained stable. That is, their displacements were zero. The mean square errors (MSEs) of plane displacements for monitoring points with different observations are shown in Table 1.

**Table 1:** Plane displacement accuracy comparison with observation correction.

Point ID	MSE without refraction(mm)	MSE with uncorrected observations(mm)	MSE with corrected observations(mm)	Distance from *R*_1_ to monitoring point(m)	Distance from *T*_1_ to monitoring point(m)
M1	6.7	26.4	7.5	120.414	661.645
M2	4.6	19.5	5.7	369.628	448.028
M3	2.6	11.2	4.2	680.600	234.378
M4	1.3	6.5	4.6	738.478	91.932
M5	3.7	13.1	4.4	700.503	359.144
M6	4.2	10.1	8.9	980.780	407.830

Table 1 indicates that the MSE of plane displacements for monitoring points calculated directly with raw observations were much larger than the theoretical situation assuming no refraction. The difference widened with the increase of the distance from the monitoring point to the station point. This proved that refraction had a significant adverse effect on the accuracy of plane displacements for monitoring points. Hence, it must be taken into consideration in actual monitoring tasks. After taking *R*_1_ as a reference to correct the raw observations towards monitoring points, the accuracy of plane displacements for monitoring points calculated with corrected observations was effectively enhanced. It appeared that the closer the monitoring point was to the reference point used for correction, the better the correction effect could be achieved, which could be caused by the similarity of the refraction effect. On the other hand, as the vertical angles from the station point to monitoring points were small in the test, the distance was the main factor affecting the correction effect. It is found from Table 1 that when the distance from the monitoring point to the station point was closer, a weaker correction quality was obtained. In this regard, when correcting the observations towards the monitoring point, a reference point closer to the monitoring point should be selected, and correction in groups can be carried out if necessary. At the same time, for effective correction, the monitoring points should not be too close to the station point when deploying the monitoring system. For height difference correction, the MSE values of vertical displacements for monitoring points with different types of observations are shown in Table 2. The table demonstrates a similar trend for plane displacements.

**Table 2:** Vertical displacement accuracy comparison with observation correction.

Point ID	MSE without refraction(mm)	MSE with uncorrected observations(mm)	MSE with corrected observations(mm)	Distance from *R*_1_ to monitoring point(m)	Distance from *T*_1_ to monitoring point(m)
M1	4.5	15.3	7.6	120.414	661.645
M2	3.1	7.8	4.4	369.628	448.028
M3	1.6	4.4	3.4	680.600	234.378
M4	0.6	2.3	2.2	738.478	91.932
M5	2.5	7.5	5.2	700.503	359.144
M6	2.8	8.9	5.9	980.780	407.830

**Fig 9:**
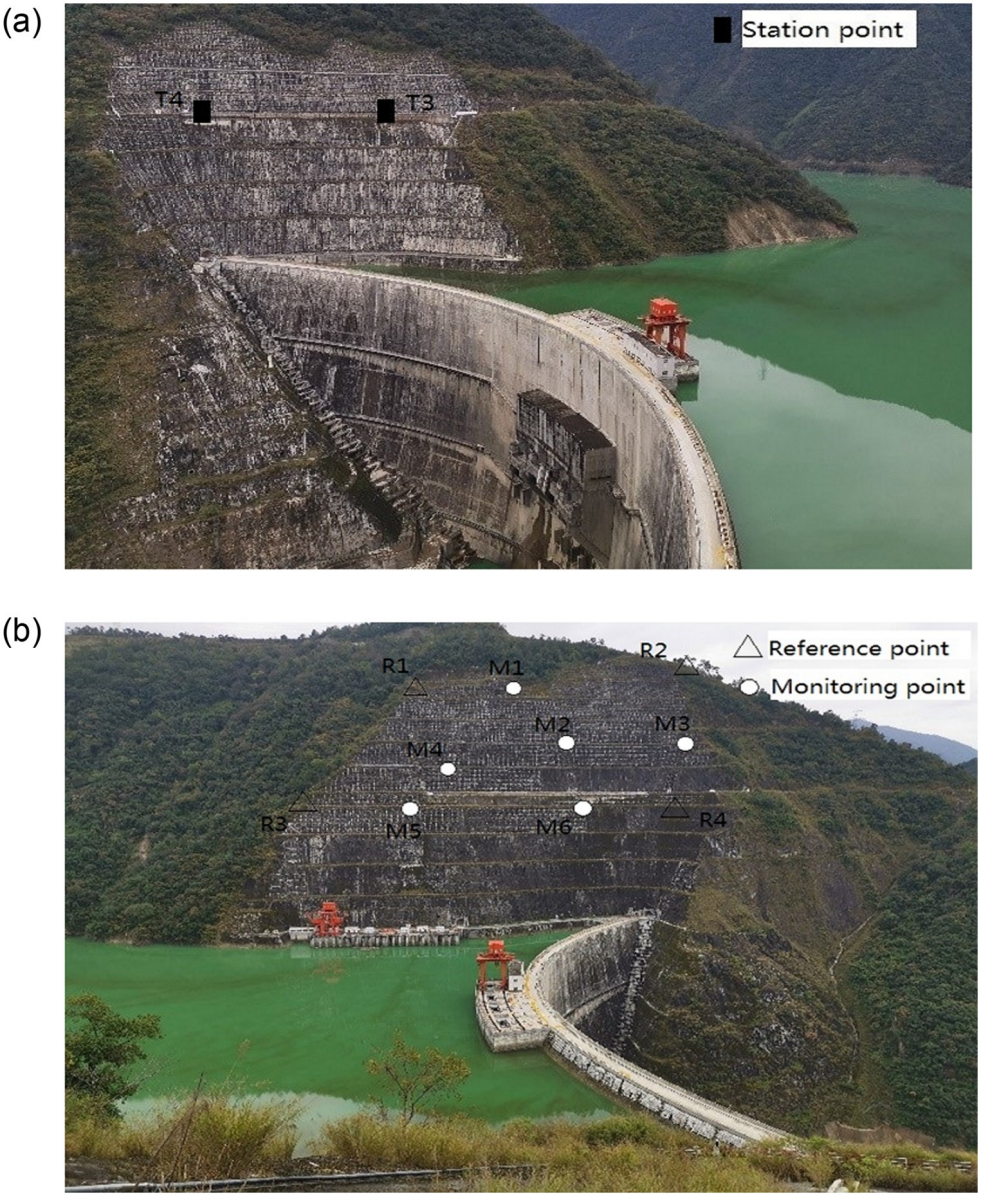
Refraction correction test in Dagangshan hydropower station. (a) Station point on the right bank. (b) Reference/monitoring points on the left bank.

The Dagangshan hydroelectric dam is a concrete double-curved arch dam located in the middle reaches of the Dadu River with a maximum dam height of 210 m. An automated monitoring system of deformation based on robotic total stations was established in 2019, and two station points were deployed on both sides of the strait to monitor slope displacement. To analyze the impact of coordinate correction for refraction, the data collected by station point *T*_3_ on the right bank was used for analysis. As shown in Fig 9(a), the robotic total station at station point *T*_3_ took station point *T*_4_ on the same bank as backsight to obtain the angle and distance observations towards the reference and monitoring points on the left bank. According to actual group observation, observations towards four stable reference points *R*_1_~*R*_4_ located around the slope of the left bank and six monitoring points *M*_1_~*M*_6_ participated in the analysis. Their distribution can be seen in Fig 9(b). A total of 23 monitoring epoch observations during approximately two weeks were utilized, of which some monitoring epochs were excluded because observations towards some points were not collected. The observations in each monitoring epoch were employed to calculate the approximate coordinates of reference and monitoring points with the polar coordinate method. Then, combined with the known coordinates of reference points, the differences in coordinates of each reference point were used to rectify the approximate coordinates of monitoring points in turns. Similarly, the monitoring points were considered stable during the monitoring period. Taking the initial coordinates of each point as the reference, The mean square errors (MSEs) of plane displacements for monitoring points under different situations are shown in Table 3.

**Table 3:** Plane displacement accuracy comparison with coordinate correction.

Point ID	MSE without refraction(mm)	MSE with uncorrected observations(mm)	MSE when corrected with *R*_1_(mm)	MSE when corrected with *R*_2_(mm)	MSE when corrected with *R*_3_(mm)	MSE when corrected with *R*_4_(mm)
M1	8.4	30.7	23.9	30.1	23.6	38.6
M2	7.9	162.1	179.9	173.5	189.6	195.1
M3	8.2	34.4	15.4	24.2	6.8	13.6
M4	7.4	23.6	5.3	15.0	7.9	18.4
M5	6.8	24.8	5.8	14.6	7.8	13.8
M6	7.0	28.4	12.9	16.9	14.5	5.2

Table 3 shows that since the distances from each monitoring point to the station point were all around 700 m, the MSE in the plane displacement for each monitoring point was around 7–8 mm in the theoretical situation assuming no refraction. When the raw observations were used to directly calculate the plane displacements of monitoring points, the MSEs increased several times compared to the theoretical situation. Overall, the plane displacement accuracy of monitoring points was improved when coordinate correction was conducted with each of the four reference points. For some monitoring points, the plane displacement accuracy after the correction was even better than the theoretical situation, such as using the reference point *R*_1_ to correct monitoring points *M*_4_ and *M*_5_. However, there was no obvious rule by which reference point could be used to produce the best correction effect. It did not seem that the closest reference point to the monitoring point could provide the best correction effect for the coordinate correction method based on the result of Table 3. Therefore, it is necessary to study how to use multiple reference points to make comprehensive corrections to the monitoring points. This is required to achieve the best correction result. Besides, the MSEs of plane displacement for monitoring point *M*_2_ under uncorrected and corrected situation greatly exceeded a certain limit. The main reason might be that the initial coordinates of the monitoring point *M*_2_ were acquired incorrectly.

## 5. Conclusion

This study mainly focused on the monitoring accuracy and refraction correction issues of continuous automatic monitoring of dam deformation based on robotic total stations. Simulation analysis showed that for the robotic total station with a given accuracy, the plane displacement accuracy of the monitoring point calculated by the forward intersection method was better than the polar coordinate method. But within a certain distance (around 400 m), the difference between the two was less than 0.5 mm, Then, for short distance monitoring, the polar coordinate method is preferred as only one total station is required, which could save the cost of the monitoring system. The distance between two total stations generally does not affect the accuracy of displacement for the forward intersection method, and it can be applied to conduct monitoring tasks with long-distance and high accuracy requirements. The actual refraction correction tests in two hydropower dams demonstrated that both the observation and coordinate corrections could effectively reduce the influence of refraction and could enhance the monitoring accuracy. For observation corrections, the closer to the reference point, the better the correction effect. Thus, it is advisable to conduct group observing according to on-site conditions. While for coordinate correction, the correction effect of a single reference point on a monitoring point lacked certain rules. Then the use of multiple reference points to achieve the better correction effect is the objective of our future research. Moreover, obtaining reliable initial coordinates of monitoring points is also crucial because it affects the accuracy of displacements.

## Supporting information

S1 File (RAR)
